# CDDO-Me Distinctly Regulates Regional Specific Astroglial Responses to Status Epilepticus via ERK1/2-Nrf2, PTEN-PI3K-AKT and NFκB Signaling Pathways

**DOI:** 10.3390/antiox9101026

**Published:** 2020-10-21

**Authors:** Ji-Eun Kim, Hana Park, Tae-Cheon Kang

**Affiliations:** 1 Department of Anatomy and Neurobiology, College of Medicine, Hallym University, Chuncheon 24252, Korea; jieunkim@hallym.ac.kr (J.-E.K.); M19050@hallym.ac.kr (H.P.); 2 Institute of Epilepsy Research, College of Medicine, Hallym University, Chuncheon 24252, Korea

**Keywords:** 3CAI, AKT, CDDO-Me, PI3K, SC79, U0126

## Abstract

2-Cyano-3,12-dioxo-oleana-1,9(11)-dien-28-oic acid methyl ester (CDDO-Me) is a triterpenoid analogue of oleanolic acid. CDDO-Me shows anti-inflammatory and neuroprotective effects. Furthermore, CDDO-Me has antioxidant properties, since it activates nuclear factor-erythroid 2-related factor 2 (Nrf2), which is a key player of redox homeostasis. In the present study, we evaluated whether CDDO-Me affects astroglial responses to status epilepticus (SE, a prolonged seizure activity) in the rat hippocampus in order to understand the underlying mechanisms of reactive astrogliosis and astroglial apoptosis. Under physiological conditions, CDDO-Me increased Nrf2 expression in the hippocampus without altering activities (phosphorylations) of phosphatase and tensin homolog deleted on chromosome 10 (*PTEN*), phosphatidylinositol-3-kinase (PI3K), and AKT. CDDO-Me did not affect seizure activity in response to pilocarpine. However, CDDO-Me ameliorated reduced astroglial Nrf2 expression in the CA1 region and the molecular layer of the dentate gyrus (ML), and attenuated reactive astrogliosis and ML astroglial apoptosis following SE. In CA1 astrocytes, CDDO-Me inhibited the PI3K/AKT pathway by activating PTEN. In contrast, CDDO-ME resulted in extracellular signal-related kinases 1/2 (ERK1/2)-mediated Nrf2 upregulation in ML astrocytes. Furthermore, CDDO-Me decreased nuclear factor-κB (NFκB) phosphorylation in both CA1 and ML astrocytes. Therefore, our findings suggest that CDDO-Me may attenuate SE-induced reactive astrogliosis and astroglial apoptosis via regulation of ERK1/2-Nrf2, PTEN-PI3K-AKT, and NFκB signaling pathways.

## 1. Introduction

Status epilepticus (SE) is an emergency neurological disorder showing prolonged seizure activities without spontaneous termination. SE has a 20–40% mortality rate and a 30% rate of neurological deficits [1]. SE leads to neuronal injury, alterations of neuronal networks, brain edema, and neuroinflammation, which cause various neurological complications, such as epilepsy and cognitive impairments [2,3,4]. SE also rapidly evokes reactive astrogliosis and astroglial apoptosis in regional specific manners independent of hemodynamics [5,6,7,8,9,10]. Reactive astrogliosis is a pathological hallmark of brain in epilepsy patients and various epilepsy models [11,12]. Although reactive astrogliosis is one of the healing processes after brain insults, it interferes with the functions of residual neuronal circuits [13]. In addition, reactive astrocyte and newly generated astrocytes after astroglial death show distinct properties, as compared to intact astrocytes, which are involved in acquisition of the physiological characteristics of the epileptic hippocampus [14,15]. This is because astrocytes maintain the brain microenvironment and homeostasis by regulating extracellular glutamate/ion concentration, brain–blood barrier, and energy metabolism [16,17]. Indeed, reactive astrogliosis and astroglial loss contribute to seizure generation via changes in responsiveness to glutamate, characteristics of Ca^2+^ oscillations, and K^+^ buffering [18,19,20,21,22]. Thus, the prevention of these astroglial responses to SE is one of the therapeutic strategies to inhibit abnormal neuronal synchronization and discharges, and the secondary undesirable post-SE complications [20,23].

2-Cyano-3,12-dioxo-oleana-1,9(11)-dien-28-oic acid methyl ester (CDDO-Me; RTA 402) is a triterpenoid analogue of oleanolic acid. CDDO-Me shows anticancer and anti-inflammatory effects. Indeed, CDDO-Me affects cellular differentiation and cell cycle arrest [24,25]. In addition, CDDO-Me has antioxidant properties, since it activates nuclear factor-erythroid 2-related factor 2 (Nrf2, a redox-sensitive transcription factor) that regulates the expression of antioxidant defense enzymes mediated by antioxidant-response element (ARE)-dependent transcription. Thus, CDDO-Me protects neurons and astrocytes from various harmful stresses including SE through the maintenance of redox homeostasis [26,27,28,29]. Furthermore, CDDO-Me distinctly affects activities of various signaling molecules, such as extracellular-signal-regulated kinase 1/2 (ERK1/2), nuclear factor-κB (NFκB), phosphatidylinositol-3-kinase (PI3K), and AKT [28,29]. Interestingly, these signaling cascades are involved in astroglial responses to SE. For example, ERK1/2 and AKT activations deteriorate reactive astrogliosis but attenuate SE-induced astroglial apoptosis. ERK1/2 and AKT also influence astroglial NFκB activity in regional specific manners following SE [30,31]. With respect to these reports, it is noteworthy to explore the effects of CDDO-Me on astroglial responses to SE and its underlying mechanisms in order to prevent post-SE complications, which has been elusive.

Here, we demonstrate that CDDO-Me effectively attenuated reactive astrogliosis in the stratum radiatum of the CA1 region (referred to as CA1 astrocytes) and astroglial apoptosis in the molecular layer of the dentate gyrus (referred to as ML astrocyte below) following SE. These effects of CDDO-Me were relevant to the regulations of phosphatase and tensin homolog deleted on the chromosome 10 (PTEN)/PI3K/AKT pathway (in CA1 astrocytes), ERK1/2-mediated Nrf2 upregulation (in ML astrocytes), and p65 RelA NFκB phosphorylations (in both CA1 and ML astrocytes). Thus, these findings suggest that CDDO-Me may inhibit the regional-specific astroglial responses to SE by regulating diverse signaling molecules.

## 2. Materials and Methods

### 2.1. Experimental Animals and Chemicals

Male Sprague–Dawley (SD) rats (7 weeks old, Daehan Biolink, South Korea) were housed under controlled temperature (22 ± 2 °C), humidity (55 ± 5%), and light/dark cycle with lights (12:12). Animals could freely access a commercial diet and water. Animal protocols were approved by the Institutional Animal Care and Use Committee of Hallym University (No. Hallym 2017-54, 19 February 2018 and Hallym 2018-2, 26 April 2018). All reagents were obtained from Sigma-Aldrich (St. Louis, MO, USA), except as noted.

### 2.2. Surgery and Chemical Infusions

Under Isoflurane anesthesia (3% induction, 1.5–2% for surgery, and 1.5% maintenance in a 65:35 mixture of N_2_O:O_2_), rats were implanted a brain infusion kit 1 (Alzet, Cupertino, CA, USA) into the right lateral ventricle (1 mm posterior; 1.5 mm lateral; −3.5 mm depth to the bregma). Thereafter, an infusion kit was connected with an Alzet 1007D osmotic pump (Alzet, Cupertino, CA, USA) containing (1) vehicle, (2) CDDO-Me (10 μM), (3) 3-chloroacetyl-indole (3CAI, an AKT inhibitor, 25 μM), (4) SC79 (an AKT activator, 25 μM), and (5) CDDO-Me + U0126 (an ERK1/2 inhibitor, 25 μM), which were continuously infused in each group over a 7-day period. Consistent with our previous studies [28,29,30], the concentration of each compound (3CAI, SC79, and U0126) did not led to behavioral and neurological abnormality in animals and alterations in seizure threshold in response to pilocarpine. The pump was placed in a subcutaneous pocket in the interscapular region. Some animals were also implanted with a monopolar stainless-steel electrode (Plastics One, Roanoke, VA, USA) into the left dorsal hippocampus (stereotaxic coordination: −3.8 mm posterior; 2.0 mm lateral; −2.6 mm depth. Electrode was secured to the exposed skull with dental acrylic. Animals were given lithium chloride (LiCl)-pilocarpine to induce SE 3 days after surgery (see below) [31,32,33].

### 2.3. SE Induction and Electroencephalogram (EEG) Analysis

Two days after surgery, rats were injected intraperitoneally (i.p.) with LiCl (127 mg/kg). Next day (three days after surgery), rats were given atropine methylbromide (5 mg/kg, i.p.) to block the peripheral effect of pilocarpine. Twenty minutes after atropine methylbromide injection, rats were administered pilocarpine (30 mg/kg, i.p., i.p.). SE induction was stopped 2 h after pilocarpine injection by treatment of diazepam (10 mg/kg, i.p.). As needed, diazepam (10 mg/kg, i.p.) was repeatedly administered. To evaluate the effect of CDDO-Me on seizure activity induced by pilocarpine, EEG signals were measured with a DAM 80 differential amplifier (0.1–3000 Hz bandpass; World Precision Instruments, Sarasota, FL, USA). EEG was acquired (400 Hz) during a 2-h recording session and analyzed using LabChart Pro v7 (AD Instruments, New South Wales, Australia). The time point starting paroxysmal discharges that showed 4–10 Hz with 2 times higher amplitude than the basal level and lasted more than 3 s was defined as the time of seizure on-set. Spectrograms were automatically made by a Hanning sliding window with a 50% overlap [28,29].

### 2.4. Tissue Processing

Three days after SE induction, animals were perfused transcardially with phosphate-buffered saline (PBS, pH 7.4) followed by 4% paraformaldehyde in 0.1 M phosphate buffer (PB, pH 7.4) under urethane anesthesia (1.5 g/kg i.p.). The brains were removed and post-fixed with the same fixative for 4 h. Subsequently, brain tissues were cryoprotected by PB containing 30% sucrose at 4 °C for 2 days. Consecutive coronal sections (30 μm) were made with a cryo-microtome and collected in six-well plates containing PBS. After decapitation under urethane anesthesia, the hippocampus was rapidly dissected out and homogenized in lysis buffer for Western blots. The protein concentration was calibrated using a Micro BCA Protein Assay Kit (Pierce Chemical, Dallas, TX, USA).

### 2.5. Immunohistochemistry and TUNEL Staining

Tissue sections were incubated with 10% normal goat serum (Vector, Burlingame, CA, USA) and subsequently with a mixture of primary antibodies in PBS containing 0.3% Triton X-100 at room temperature, overnight (Table 1). After washing, tissues were reacted with a fluorescein isothiocyanate (FITC) or Cy3-conjugated secondary antibodies (Vector, Burlingame, CA, USA) for 1 h at room temperature. For negative control, sections were reacted with pre-immune serum instead of primary antibody. An AxioImage M2 microscope and AxioVision Rel. 4.8 software (Carl Zeiss Korea, Seoul, Korea) were used for image capture and analysis of fluorescent intensity. Fluorescent intensity was measured and represented as the number of a 256-gray scale (15 sections per each animal). The intensities were corrected by subtracting the average values of background noise obtained from 5 image inputs. To analyze the astroglial apoptosis, terminal deoxynucleotidyl transferase dUTP nick end labeling (TUNEL) staining was applied according to the manufacturer’s instructions (Upstate, Lake Placid, NY, USA) prior to immunofluorescent study. Areas of interest in the dentate gyrus (1 × 10^5^ μm^2^, 15 sections per each animal) were selected, and cell count of TUNEL-positive cells was performed using AxioVision Rel. 4.8 software.

### 2.6. Western Blot

After electrophoresis and transfer, membranes were blocked overnight at 4 °C with 2% bovine serum albumin (BSA) in Tris-buffered saline (TBS; in mM 10 Tris, 150 NaCl, pH 7.5, and 0.05% Tween 20) and subsequently incubated in primary antibodies (Table 1). After washing, membranes were incubated for 1 h at room temperature in a solution containing horseradish peroxidase (HRP)-conjugated secondary antibodies. Immunoblots were quantified by membrane scanning in an ImageQuant LAS4000 system (GE Healthcare Korea, Seoul, South Korea). Optical densities of proteins were measured by the protein/β-actin ratio. The ratio of phosphoprotein to total protein was described as the phosphorylation level.

### 2.7. Quantification of Data and Statistical Analysis

The number (*n*) of each experimental group used for the evaluation was seven. The data obtained from each group were analyzed. After evaluating the values on normality using Shapiro–Wilk *W*-test, all results were analyzed by the two-tailed Student *t*-test, repeated one-way ANOVA, or one-way ANOVA to determine statistical significance. The Newman–Keuls test was used for post hoc comparisons. A *p*-value below 0.05 was considered statistically significant.

## 3. Results

### 3.1. CDDO-Me Increases Nrf2 Expression and Attenuates Reactive CA1 Astrogliosis and Apoptosis of ML Astrocytes Following SE

**Figure 1:**
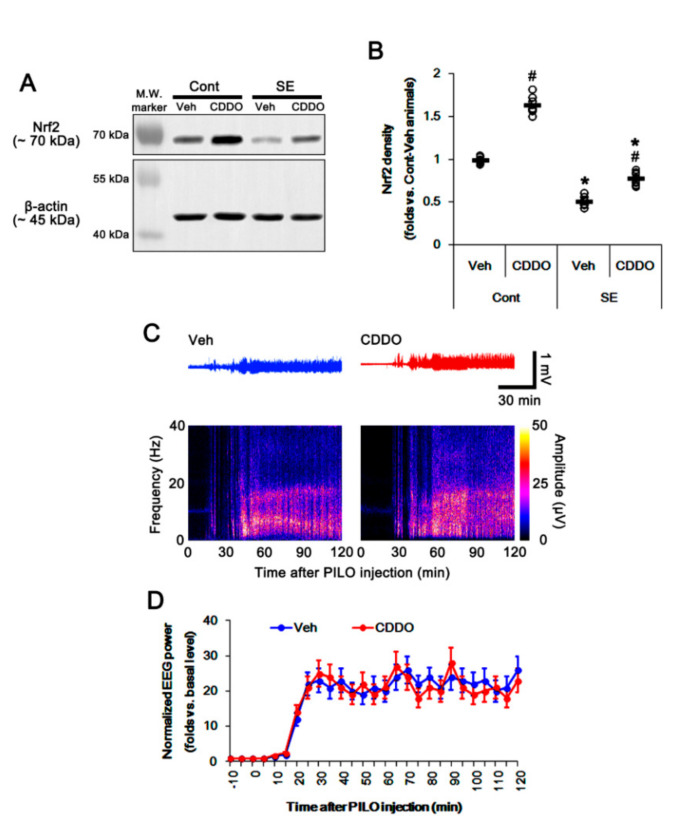
Effects of 2-Cyano-3,12-dioxo-oleana-1,9(11)-dien-28-oic acid methyl ester (CDDO-Me) on nuclear factor-erythroid 2-related factor 2 (Nrf2) expression and seizure activity in response to pilocarpine. (**A**,**B**) Effect of CDDO-Me on Nrf2 expression in response to pilocarpine. CDDO-Me increases Nrf2 expression under physiological conditions. Pilocarpine-induced status epilepticus (SE) decreases Nrf2 expression, which is attenuated by CDDO-Me. (**A**) Representative Western blots of Nrf2 expression. (**B**) Quantification of Nrf2 expression based on Western blot data. Open circles indicate each individual value. Horizontal and vertical bars indicate mean value and S.E.M., respectively (mean ± S.E.M.; *,# *p* < 0.05 vs. control animals and vehicle, respectively, one-way ANOVA followed by Newman–Keuls post hoc test; n = 7, respectively). (**C**,**D**) Effect of CDDO-Me on seizure activity in response to pilocarpine. CDDO-Me does not influence seizure activity induced by pilocarpine. (**C**) Representative electroencephalogram (EEG) traces and frequency-power spectral temporal maps in response to pilocarpine. (**D**) Quantification of total EEG power (seizure intensity) in response to pilocarpine. Open circles and vertical bars indicate mean value and S.E.M., respectively (mean ± S.E.M.; repeated one-way ANOVA; n = 7, respectively).

Under physiological conditions, CDDO-Me increased Nrf2 expression in the whole hippocampus (*p* < 0.05 vs. vehicle, one-way ANOVA followed by Newman–Keuls post hoc test; n = 7, respectively; Figure 1A,B and Supplementary Figure S1). Consistent with our previous study [28], CDDO-Me did not affect the seizure latency and its severity in response to pilocarpine (repeated one-way ANOVA; n = 7; Figure 1C,D). Following SE, total Nrf2 expression was decreased in the whole hippocampus (*p* < 0.05 vs. vehicle, one-way ANOVA followed by Newman–Keuls post hoc test; n = 7, respectively; Figure 1A,B and Supplementary Figure S1).

**Figure 2:**
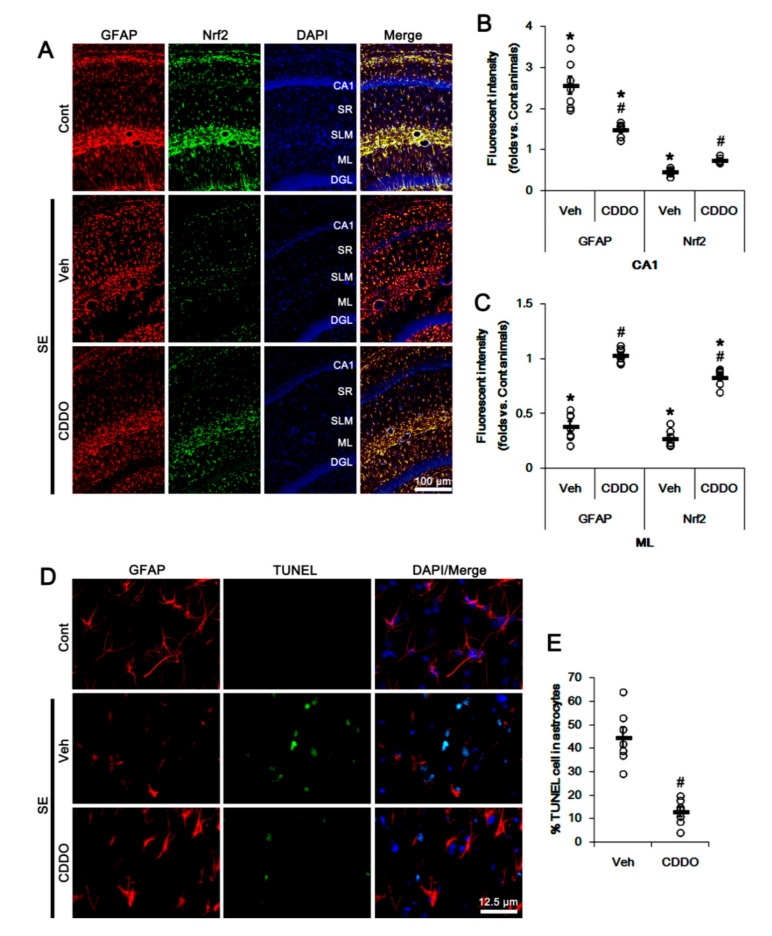
Effects of CDDO-Me on Nrf2 expression and astroglial responses following SE. CDDO-Me ameliorates the reduced Nrf2 expression in CA1 astrocytes and ML astrocytes following SE. CDDO-Me also attenuates reactive CA1 astrogliosis and ML astroglial apoptosis induced by SE. (**A**) Representative photos of GFAP and Nrf2 in the hippocampus. Abbreviations: CA1, CA1 pyramidal cell layer; SR, stratum radiatum; SLM, stratum lacunosum-moleculare; ML, molecular layer of the dentate gyrus; DGL, dentate granule cell layer. (**B**,**C**) Quantification of the fluorescent intensities of GFAP and Nrf2 in CA1 (**B**) and molecular layer regions (**C**). Open circles indicate each individual value. Horizontal bars indicate mean value. Vertical bars indicate S.E.M. (*,# *p* < 0.05 vs. control animals and vehicle, respectively, one-way ANOVA followed by Newman-Keuls post hoc test; n = 7, respectively). (**D**) Representative photos of apoptosis of ML astrocytes. (**E**) Quantification of the fraction of TUNEL-signals in ML astrocytes. Open circles indicate each individual value. Horizontal bars indicate mean value. Vertical bars indicate S.E.M. (# *p* < 0.05 vs. vehicle, two-tailed Student *t*-test; n = 7, respectively).

In addition, CA1 astrocytes showed hypertrophy and upregulated GFAP expression (reactive astrogliosis; *p* < 0.05 vs. control animals, one-way ANOVA followed by Newman-Keuls post hoc test; n = 7, respectively; Figure 2A,B). In addition, TUNEL staining showed SE-induced apoptosis of ML astrocytes (*p* < 0.05 vs. control animals, one-way ANOVA followed by Newman-Keuls post hoc test; n = 7, respectively; Figure 2A,C–E). As compared to control animals, Nrf2 expression was reduced in reactive CA1 astrocytes and remaining ML astrocytes (*p* < 0.05 vs. control animals, one-way ANOVA followed by Newman-Keuls post hoc test; n = 7, respectively; Figure 2A–C). CDDO-Me effectively ameliorated the reduced Nrf2 expression in CA1 and ML astrocytes, and attenuated reactive CA1 astrogliosis and ML astroglial apoptosis following SE (*p* < 0.05 vs. vehicle, one-way ANOVA followed by Newman-Keuls post hoc test and two-tailed Student *t*-test; n = 7, respectively; Figure 2A–E). These findings suggest that CDDO-Me may upregulate Nrf2 expression and prevent reactive CA1 astrogliosis as well as astroglial degeneration following SE.

### 3.2. CDDO-Me Ameliorates Reactive CA1 Astrogliosis by Regulating the PTEN/PI3K/AKT Pathway

**Figure 3:**
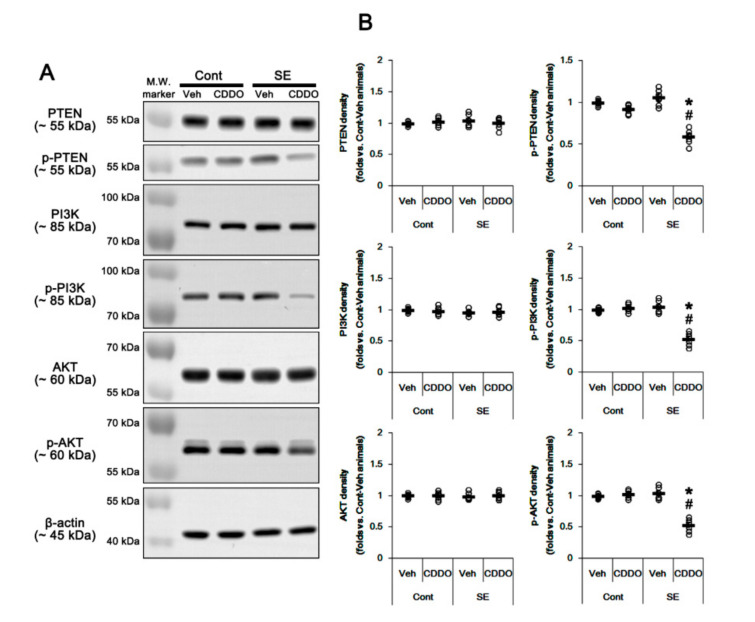
Effects of CDDO-Me on activities (phosphorylations) of PTEN, PI3K, and AKT following SE. PTEN, PI3K, and AKT expressions and their phosphorylations are unaffected by SE. CDDO-Me reduces PTEN, PI3K, and AKT phosphorylations without changing their expression levels under the post-SE condition. (**A**) Representative Western blots of PTEN, PI3K, and AKT expressions and their phosphorylations. (**B**) Quantification of PTEN, PI3K, and AKT expressions and their phosphorylations based on Western blot data. Open circles indicate each individual value. Horizontal and vertical bars indicate mean value and S.E.M., respectively (mean ± S.E.M.; *,# *p* < 0.05 vs. control animals and vehicle, respectively, one-way ANOVA followed by Newman-Keuls post hoc test; n = 7, respectively).

Nrf2 expression and its activity are regulated by the PI3K/AKT signaling pathway [34,35]. Therefore, we investigated whether CDDO-Me mitigates astroglial responses by influencing this pathway. In the whole hippocampus, CDDO-Me did not influence expressions and phosphorylations of PI3K and AKT under physiological condition, which were unaffected by SE (one-way ANOVA followed by Newman-Keuls post hoc test; n = 7, respectively; Figure 3A,B and Supplementary Figure S1). Following SE, CDDO-Me reduced phosphorylation levels of PI3K/AKT in the whole hippocampus without altering their expression levels (*p* < 0.05 vs. control animals and vehicle, one-way ANOVA followed by Newman-Keuls post hoc test; n = 7, respectively; Figure 3A,B and Supplementary Figure S1). These findings indicate that CDDO-Me may inhibit PI3K/AKT signaling cascades.

Since PTEN negatively regulates the PI3K/AKT pathway [36], we also validated the effect of CDDO-Me on PTEN. SE did not affect PTEN expression and its phosphorylation (one-way ANOVA followed by Newman-Keuls post hoc test; n = 7, respectively; Figure 3A,B and Supplementary Figure S1). However, CDDO-Me reduced PTEN phosphorylation without changing its expression level under the post-SE condition but not the physiological condition (*p* < 0.05 vs. control animals and vehicle, one-way ANOVA followed by Newman-Keuls post hoc test; n = 7, respectively; Figure 3A,B). Considering that PTEN phosphorylation represents its inactivation [37], these findings indicate that CDDO-Me may inhibit the PI3K/AKT pathway by increasing PTEN activity under the post-SE condition.

**Figure 4:**
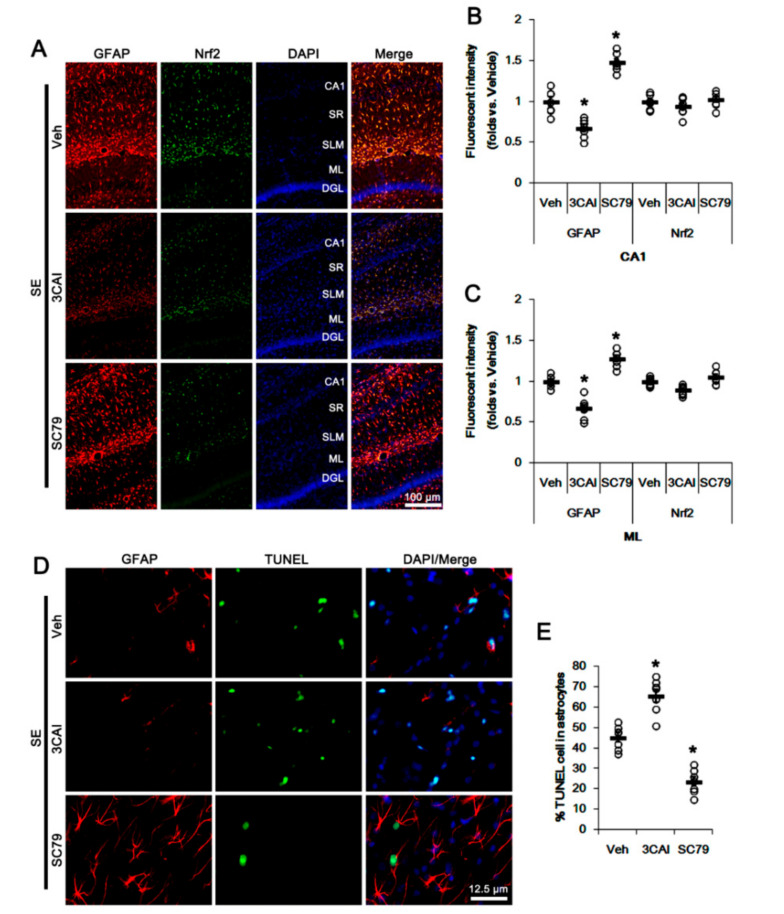
Effects of 3CAI and SC79 on Nrf2 expression and astroglial responses following SE. 3CAI attenuates reactive CA1 astrogliosis but deteriorates ML astroglial apoptosis, which are reversed by SC79. Neither 3CAI nor SC79 affects Nrf2 expression in CA1 astrocytes and ML astrocytes following SE. (**A**) Representative photos of GFAP and Nrf2 in the hippocampus. Abbreviations: CA1, CA1 pyramidal cell layer; SR, stratum radiatum; SLM, stratum lacunosum-moleculare; ML, molecular layer of the dentate gyrus; DGL, dentate granule cell layer. (**B**,**C**) Quantification of the fluorescent intensities of GFAP and Nrf2 in CA1 (**B**) and molecular layer regions (**C**). Open circles indicate each individual value. Horizontal bars indicate mean value. Vertical bars indicate S.E.M. (* *p* < 0.05 vs. vehicle, one-way ANOVA followed by Newman-Keuls post hoc test; n = 7, respectively). (**D**) Representative photos of apoptosis of ML astrocytes. (**E**) Quantification of the fraction of TUNEL-signals in ML astrocytes. Open circles indicate each individual value. Horizontal bars indicate mean value. Vertical bars indicate S.E.M. (* *p* < 0.05 vs. vehicle, one-way ANOVA followed by Newman-Keuls post hoc test; n = 7, respectively).

To further confirm the roles of AKT in Nrf2 expression and astroglial responses following SE, we applied 3CAI (an AKT inhibitor) and SC79 (an AKT activator) prior to SE induction. Consistent with our previous study [31], 3CAI attenuated reactive CA1 astrogliosis but deteriorated ML astroglial apoptosis following SE, which were reversed by SC79 (*p* < 0.05 vs. vehicle, one-way ANOVA followed by Newman-Keuls post hoc test; n = 7, respectively; Figure 4A–E). However, neither 3CAI and nor SC79 changed astroglial Nrf2 expression (one-way ANOVA followed by Newman-Keuls post hoc test; n = 7, respectively; Figure 4A–C). These findings indicate that CDDO-Me-mediated PI3K/AKT inhibition may attenuate SE-induced reactive CA1 astrogliosis, although this pathway may not be involved in ML astroglial apoptosis and upregulation of Nrf2 expression.

### 3.3. CDDO-Me Attenuates Reactive CA1 Astrogliosis and Astroglial Apoptosis by Inhibiting NFκB Phosphorylation

**Figure 5:**
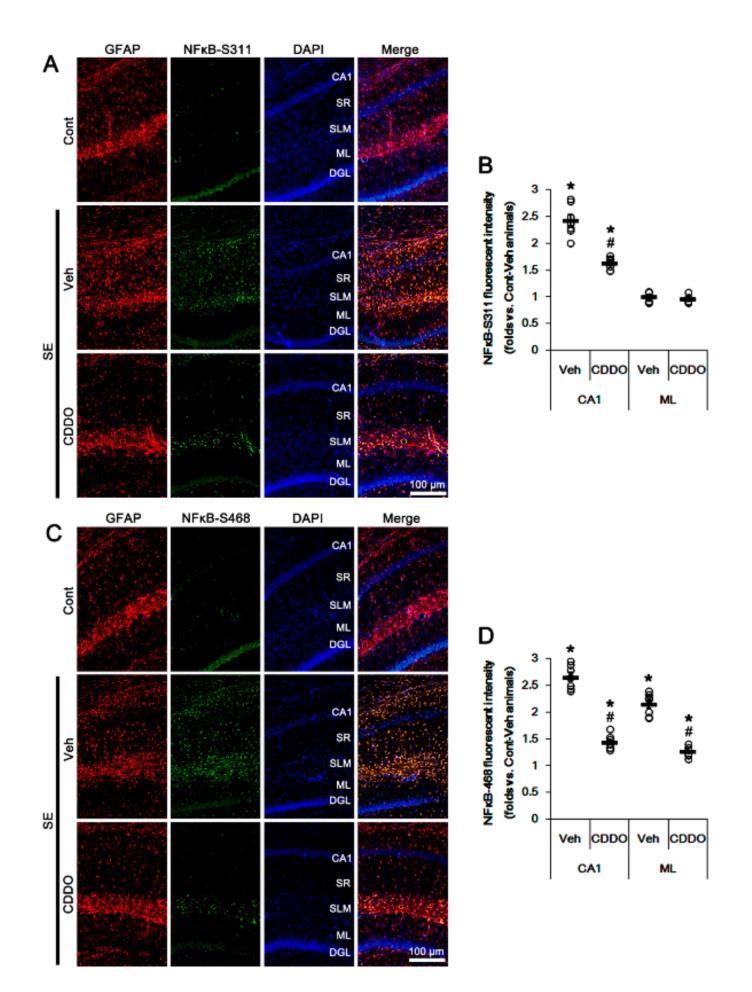
Effects of CDDO-Me on NFκB phosphorylation following SE. NFκB-S311 and -S468 phosphorylations are increased in reactive CA1 astrocytes, but only-S468 phosphorylation is elevated in ML astrocytes, which were abrogated by CDDO-Me. (**A**) Representative photos of NFκB-S311 phosphorylation in the hippocampus. Abbreviations: CA1, CA1 pyramidal cell layer; SR, stratum radiatum; SLM, stratum lacunosum-moleculare; ML, molecular layer of the dentate gyrus; DGL, dentate granule cell layer. (**B**) Quantification of the fluorescent intensity of NFκB-S311 phosphorylation in CA1 and molecular layer regions. Open circles indicate each individual value. Horizontal bars indicate mean value. Vertical bars indicate S.E.M. (*,# *p* < 0.05 vs. control animals and vehicle, respectively, one-way ANOVA followed by Newman-Keuls post hoc test; n = 7, respectively). (**C**) Representative photos of NFκB-S468 phosphorylation in the hippocampus. (**D**) Quantification of the fluorescent intensity of NFκB-S468 phosphorylation in CA1 and molecular layer regions. Open circles indicate each individual value. Horizontal bars indicate mean value. Vertical bars indicate S.E.M. (*,# *p* < 0.05 vs. control animals and vehicle, respectively, one-way ANOVA followed by Newman-Keuls post hoc test; n = 7, respectively).

NFκB phosphorylation plays an important role in the maintenance of its optimal activity [38]. In particular, NFκB S311 phosphorylation increases its transcriptional activity and anti-apoptotic function [39]. In contrast, S468 phosphorylation terminates NFκB-dependent gene expression upon assisting in binding of an E3 ubiquitin ligase complex to NFκB [40]. In addition, reactive CA1 astrogliosis and ML astroglial apoptosis are differently regulated by NFκB phosphorylation following SE: NFκB-S311 phosphorylation modulates reactive CA1 astrogliosis, while its S468 phosphorylation is involved in ML astroglial apoptosis [31,41]. Since CDDO-Me directly inhibits NFκB activity [24,42,43,44], it is likely that CDDO-Me may attenuate reactive CA1 astrogliosis and ML astroglial apoptosis by inhibiting NFκB phosphorylation. In the present study, SE increased NFκB-S311 and S468 phosphorylation in reactive CA1 astrocytes but only-S468 phosphorylation in ML astrocytes, which were abrogated by CDDO-Me (*p* < 0.05 vs. control animals and vehicle, one-way ANOVA followed by Newman-Keuls post hoc test; n = 7, respectively; Figure 5A–D). Therefore, our findings suggest that CDDO-Me may ameliorate SE-induced astroglial responses by inhibiting NFκB phosphorylation.

### 3.4. CDDO-Me Increases Nrf2 Expression by Enhancing ERK1/2 Activity

**Figure 6:**
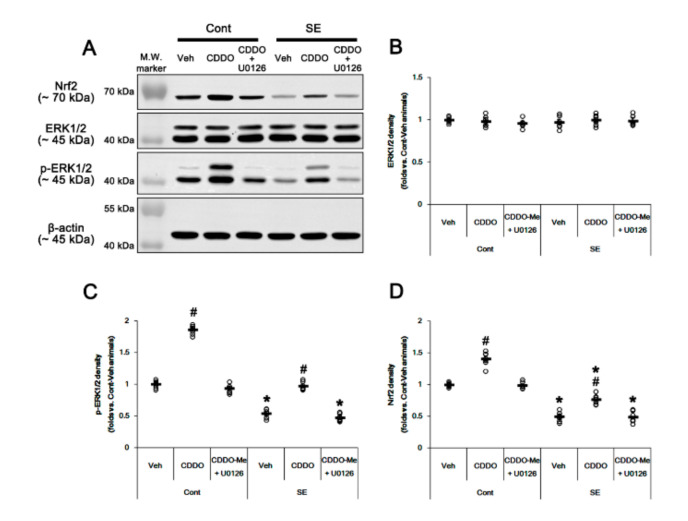
Effects of CDDO-Me and U0126 co-treatment on Nrf2 expression and ERK1/2 activity (phosphorylation) following SE. Under physiological conditions, CDDO-Me increases ERK1/2 phosphorylation and Nrf2 expression. CDDO-Me also attenuates SE-induced reductions in ERK1/2 phosphorylation and Nrf2 expression. U0126 co-treatment abolishes the CDDO-Me-induced upregulation of ERK1/2 phosphorylation and Nrf2 expression under physiological and post-SE conditions. (**A**) Representative Western blots of Nrf2, ERK1/2, and p-ERK1/2. (**B**,**D**) Quantification of ERK1/2 (**B**), p-ERK1/2 (**C**), and Nrf2 (**D**) levels based on Western blot data. Open circles indicate each individual value. Horizontal and vertical bars indicate mean value and S.E.M., respectively (mean ± S.E.M.; *,# *p* < 0.05 vs. control animals and vehicle, respectively, one-way ANOVA followed by Newman-Keuls post hoc test; n = 7).

**Figure 7:**
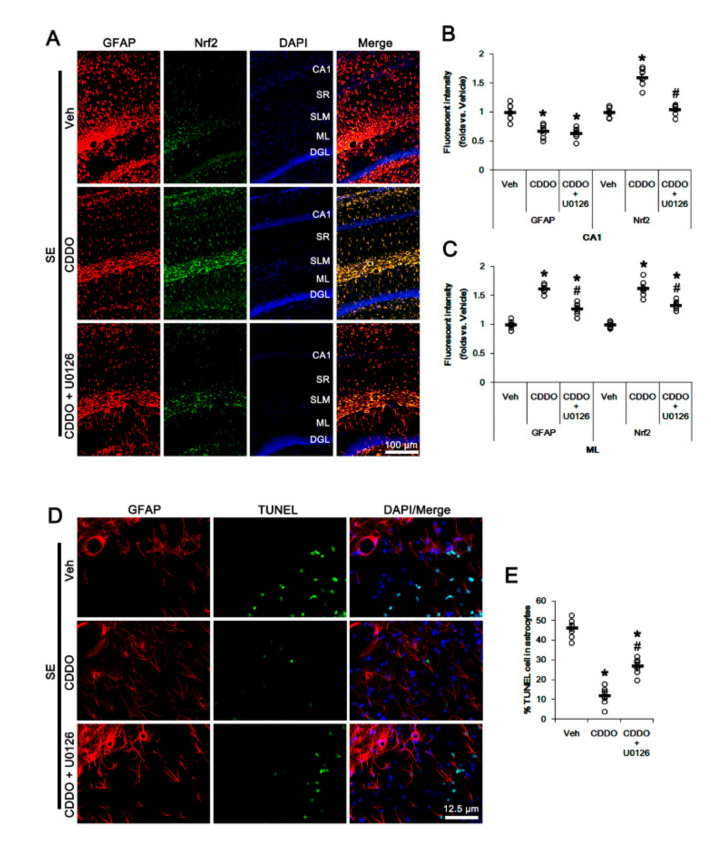
Effects of CDDO-Me and U0126 co-treatment on Nrf2 expression and astroglial responses following SE. U0126 co-treatment does not affect the abolishment of reactive CA1 astrogliosis by CDDO-Me. However, U0126 co-treatment inhibits the upregulation of Nrf2 expression induced by CDDO-Me in CA1 astrocytes and ML astrocytes. In addition, U0126 abrogates the protective effect of CDDO-Me against SE-induced ML astroglial apoptosis. (**A**) Representative photos of GFAP and Nrf2 in the hippocampus. Abbreviations: CA1, CA1 pyramidal cell layer; SR, stratum radiatum; SLM, stratum lacunosum-moleculare; ML, molecular layer of the dentate gyrus; DGL, dentate granule cell layer. (**B**,**C**) Quantification of the fluorescent intensities of GFAP and Nrf2 in CA1 (**B**) and molecular layer regions (**C**). Open circles indicate each individual value. Horizontal bars indicate mean value. Vertical bars indicate S.E.M. (*,# *p* < 0.05 vs. vehicle and CDDO-Me, respectively, one-way ANOVA followed by Newman-Keuls post hoc test; n = 7, respectively). (**D**) Representative photos of apoptosis of ML astrocytes. (**E**) Quantification of the fraction of TUNEL-signals in ML astrocytes. Open circles indicate each individual value. Horizontal bars indicate mean value. Vertical bars indicate S.E.M. (*,# *p* < 0.05 vs. vehicle and CDDO-Me, respectively, one-way ANOVA followed by Newman-Keuls post hoc test; n = 7, respectively).

Since CDDO-Me increases ERK1/2 activity [25,28,45], which stimulates the Nrf2 system in astrocytes [46], we evaluated whether ERK1/2 is one of the upstream effectors of CDDO-Me-mediated upregulation of Nrf2. In the present study, SE significantly reduced the ERK1/2 phosphorylation level without changing its expression (*p* < 0.05 vs. control animals, one-way ANOVA followed by Newman-Keuls post hoc test; n = 7, respectively; Figure 6A–C and Supplementary Figure S2). Under physiological conditions, CDDO-Me increased the ERK1/2 phosphorylation level (*p* < 0.05 vs. vehicle, one-way ANOVA followed by Newman-Keuls post hoc test; n = 7, respectively; Figure 6A–C and Supplementary Figure S2). Following SE, CDDO-Me attenuated reductions in ERK1/2 phosphorylation and Nrf2 expression (*p* < 0.05 vs. vehicle, one-way ANOVA followed by Newman-Keuls post hoc test; n = 7, respectively; Figure 6A–D and Supplementary Figure S2). Co-treatment of U0126 (an ERK1/2 inhibitor) abolished the CDDO-Me-induced upregulation of ERK1/2 phosphorylation and Nrf2 expression under physiological and post-SE conditions (*p* < 0.05 vs. CDDO-Me, one-way ANOVA followed by Newman-Keuls post hoc test; n = 7, respectively; Figure 6A,D and Supplementary Figure S2). U0126 co-treatment did not affect the abolishment of reactive CA1 astrogliosis by CDDO-Me (one-way ANOVA followed by Newman-Keuls post hoc test; n = 7, respectively; Figure 7A–B). However, U0126 co-treatment inhibited the upregulation of Nrf2 expression induced by CDDO-Me in CA1 astrocytes and ML astrocytes (*p* < 0.05 vs. CDDO-Me, one-way ANOVA followed by Newman-Keuls post hoc test; n = 7, respectively; Figure 7A–C). In addition, U0126 blocked the protective effect of CDDO-Me against SE-induced ML astroglial apoptosis (*p* < 0.05 vs. vehicle and CDDO-Me, one-way ANOVA followed by Newman-Keuls post hoc test; n = 7, respectively; Figure 7D,E). These findings indicate that CDDO-Me-induced ERK1/2 activation may protect ML astrocytes from SE by increasing Nrf2 expression, which may not affect reactive CA1 astrogliosis.

## 4. Discussion

Following brain insults, astrocytes show hypertrophy and proliferation in the affected region, which is termed reactive astrogliosis [47]. Reactive astrogliosis is one of the scar tissues that inhibit dendritic and axonal remodeling in neuronal circuits. Reactive astrocytes also influence neural viability and synaptogenesis after brain injury by releases of growth factors and trophic factors [48,49]. SE-induced reactive astrogliosis originates from distinct sources and different pathways in the hippocampus: gliogenesis in the dentate gyrus and in situ proliferation in the stratum radiatum of the CA1 region [5,14]. Thus, the mechanisms that regulate reactive astrogliosis are complex and remain elusive. Recently, we reported that PI3K/AKT inhibition attenuates reactive CA1 astrogliosis following SE [31]. In the present study, CDDO-Me abolished SE-induced reactive CA1 astrogliosis concomitant with reduced PI3K/AKT phosphorylation. These findings are consistent with previous reports demonstrating CDDO-Me-induced AKT inhibition [50,51,52,53]. Furthermore, our findings demonstrate that CDDO-Me-activated (dephosphorylated) PTEN, which leads to the constitutive PI3K/AKT inhibition [54]. The present data also reveal that 3CAI (an AKT inhibitor) also inhibited reactive CA1 astrogliosis, which were reversed by SC79 (an AKT activator). Similar to 3CAI, U0126 attenuated reactive CA1 astrogliosis [31]. In the present study, however, U0126 co-treatment did not enhance the inhibitory effect of CDDO-ME on reactive CA1 astrogliosis. Since the PI3K/AKT pathway plays an important role in regulating cell division and viability [55,56], these findings indicate that CDDO-Me may mitigate reactive CA1 astrogliosis through the PTEN/PI3K/AKT system rather than the ERK1/2 pathway.

On the other hand, neuronal death is one of the powerful signals that induces reactive astrogliosis [57,58]. Since CDDO-Me selectively attenuates CA1 pyramidal cell loss induced by SE [28], it cannot be excluded the possibility that abrogation of reactive CA1 astrogliosis would result from the neuroprotective effect of CDDO-Me. However, recent studies reveal that reactive astrogliosis is irrelevant to neuronal death [30,32,33]. Furthermore, AKT inhibition does not affect CA1 neuronal death induced by SE [30], and CDDO-Me did not affect the PI3K/AKT pathway under physiological condition in the present study. Therefore, our findings indicate that CDDO-Me may ameliorate reactive CA1 astrogliosis by regulating the PTEN/PI3K/AKT signaling pathway, independent of CA1 neuronal loss.

SE and chronic epileptic seizure activity induce astroglial loss in the epileptic patient and animal models [5,59,60]. Consistent with our previous study [31], the present study demonstrates that 3CAI (an AKT inhibitor) aggravated SE-induced ML astroglial apoptosis, while SC79 (an AKT activator) alleviated it following SE. Since the PI3K/AKT pathway maintains cellular viability and inhibits apoptosis [55,56,61], PI3K/AKT activation may play a pro-survival role in astrocytes against apoptosis. In the present study, however, CDDO-Me ameliorated ML astroglial apoptosis induced by SE, although it inhibited PI3K/AKT activities. Thus, it is likely that PI3K/AKT inhibition by CDDO-Me may regulate astroglial activation without altering its viability. Similar to 3CAI, U0126 deteriorates SE-induced ML astroglial loss [31]. SE induces ML astroglial degeneration by caspase-3-independent apoptosis-inducing factor (AIF)-mediated apoptosis [5,8,62], which is evoked by oxidative stress [63]. In the present study, CDDO-Me increased astroglial Nrf2 expression under physiological and post-SE conditions. In addition, co-treatment of U0126 (an ERK1/2 inhibitor) inhibited CDDO-Me-induced Nrf2 upregulation under physiological and post-SE conditions. Thus, these findings indicate that CDDO-Me may protect ML astrocytes from SE-induced oxidative damage through ERK1/2-mediated Nrf2 induction. Indeed, CDDO and its derivatives activate the Nrf2-ARE pathway by dissociating Nrf2 from Kelch-like ECH-associated protein 1 (Keap1) in the cytoplasm, which results in the synthesis of many antioxidant and phase II detoxifying enzymes, including catalase, heme oxygenase 1, and enzymes involved in glutathione (GSH) production [42,64,65,66,67]. Furthermore, CDDO and its derivatives activate the Nrf2-ARE pathway in astrocytes rather than neurons. This is because the Nrf2-ARE pathway shows a disparity between astrocytes and neurons [68,69], and neurons have lower basal levels of Nrf2 relative to astrocytes [70]. Considering PI3K/AKT-independent ERK1/2-mediated Nrf2 regulation and astroglial survival [25,29,31,32,34,35,45,46], our findings suggest that CDDO-Me may protect astrocyte against SE by upregulation of Nrf2 expression via the ERK1/2 pathway rather than the PI3K/AKT system.

NFκB phosphorylation enhances the transactivation of several anti-apoptotic and pro-survival genes. Indeed, NFκB S311 phosphorylation increases its transcriptional activity and anti-apoptotic function [39], while S468 phosphorylation terminates NFκB-dependent gene expression upon assisting in binding of an E3 ubiquitin ligase complex to NFκB [40]. In addition, muscarinic receptor (receptor for pilocarpine) activation directly modulates NFκB phosphorylation in astrocytes [71]. Consistent with our previous studies [31,41], the present study shows that SE increased NFκB-S311 and-S468 phosphorylation in reactive CA1 astrocytes but only-S468 phosphorylation in the ML astrocytes. CDDO-Me diminished the upregulation of NFκB-S311 and-S468 phosphorylation induced by SE. In a previous study [31], we found that AKT or ERK1/2 inhibition attenuates SE-induced NFκB S311 and S468 phosphorylation during reactive astrogliosis, while they increased these NFκB S468 phosphorylation in ML astrocytes. Considering the effect of CDDO-Me on AKT and ERK1/2 activities in the present study, it is likely that CDDO-Me may inhibit NFκB phosphorylation independent of the AKT and ERK1/2 signaling pathways. Indeed, CDDO-Me directly inhibits NFκB activity [24,42,43,44]. Furthermore, N-(2-cyano-3,12-dioxo-28-noroleana-1,9(11)-dien-17-yl)-2-2-difluoropropanamide (RTA 408), another CDDO derivative, attenuates reactive astrogliosis by suppression of AKT activation and NFκB phosphorylation [72], although AKT inhibits NFκB-S468 phosphorylation [73]. Thus, CDDO-Me may also mitigate reactive CA1 astrogliosis and ML astroglial apoptosis through direct NFκB inhibition. Recently, Meng et al. [74] also reported that CDDO-imidazolide (CDDO-Im) enhances Nrf2-mediated antioxidant and anti-inflammatory activity by reacting with thiols on Keap1. Unlike CDDO-Me, CDDO-Im can covalently bind to arginine and serine residues other than cysteine on target proteins. In addition, CDDO-Im binds to Keap1 by composing permanent Michael adducts with eight different cysteines, and acyl adducts with lysine and tyrosine residues, which enhance the potency of CDDO-Im. Considering these reports, it will be needed to explore the potential efficacies and cellular signaling pathways of other triterpenoids in reactive astrogliosis and astroglial apoptosis.

## 5. Conclusions

**Figure 8:**
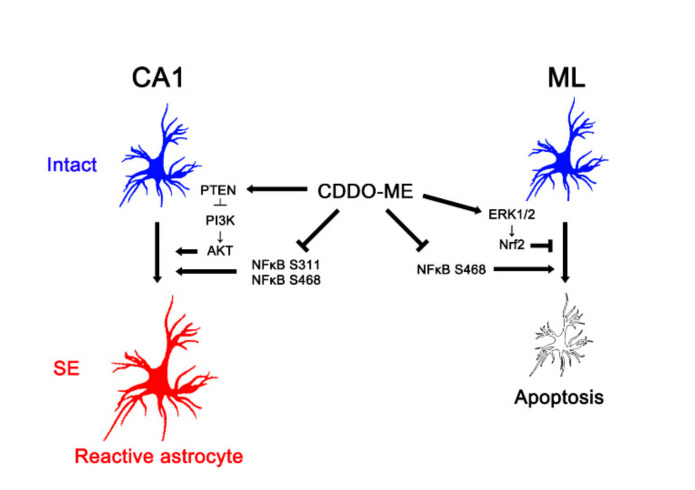
Scheme of the effects of CDDO-Me on reactive CA1 astrogliosis and ML astroglial apoptosis. CDDO-Me attenuates reactive CA1 astrogliosis by activating PTEN, which inhibits PI3K/AKT activities. CDDO-Me also abates reactive CA1 astrogliosis by reducing NFκB S311 and S468 phosphorylation. In addition, CDDO-Me ameliorates ML astroglial apoptosis via upregulation of ERK1/2-mediated Nrf2 expression and inhibition of NFκB S468 phosphorylation. Therefore, CDDO-Me mitigates both reactive astrogliosis and astroglial degeneration induced by SE.

In conclusion, the present study reports a novel effect of CDDO-Me on reactive astrogliosis and astroglial apoptosis induced by SE, and demonstrates its underlying molecular mechanisms mediated by the PTEN/PI3K/AKT, ERK1/2, and NFκB signaling pathways. Briefly, CDDO-Me ameliorated reactive CA1 astrogliosis by enhancing PTEN-mediated PI3K/AKT inhibition. In addition, it attenuated ML astroglial apoptosis via an increase of ERK1/2-mediated Nrf2 upregulation. CDDO-Me also abated reactive astrogliosis and astroglial apoptosis by abolishing NFκB phosphorylation (Figure 8). Therefore, our findings propose the availability of CDDO-Me and its derivates for various neurological diseases relating to reactive astrogliosis and astroglial degeneration.

## Figures and Tables

**Table 1:** Primary antibodies used in the present study.

Antigen	Host	Manufacturer (Catalog Number)	Dilution Used
AKT	Rabbit	Cell signaling (#9272)	1:1000 (WB)
ERK1/2	Rabbit	Biorbyt (Orb160960)	1:1000 (WB)
GFAP	Mouse	Millipore (#MAB3402)	1:1000 (IH)
NF-κB RelA p65-S311	Rabbit	Abcam (ab194926)	1:100 (IH)
NF-κB RelA p65-S468	Rabbit	Abcam (ab31473)	1:100 (IH)
Nrf2	mouse	Abcam (ab89443)	1:1000 (WB) 1:100 (IH)
p-AKT-S473	Rabbit	Cell signaling (#4060)	1:1000 (WB)
p-ERK1/2	Rabbit	Millipore (#05-797RSP)	1:1000 (WB)
p-PI3K-Y458	Rabbit	Cell signaling (#4228S)	1:1000 (WB)
p-PTEN	Rabbit	Cell signaling (#9549)	1:1000 (WB)
PI3K	Rabbit	Cell signaling (#4292S)	1:1000 (WB)
PTEN	Rabbit	Abcam (ab32199)	1:5000 (WB)
β-actin	Mouse	Sigma (#A5316)	1:5000 (WB)

IH: Immunohistochemistry; WB: Western blot.

## Supplementary Materials

The following are available online at https://www.mdpi.com/2076-3921/9/10/1026/s1, Figure S1: Full-gel images of Western blots in Figure 1A and Figure 3A, Figure S2: Full-gel images of Western blots in Figure 6A.

## References

[1] Shorvon S., Sen A. (2020). What is status epilepticus and what do we know about its epidemiology?. Seizure.

[2] Reddy D.S., Kuruba R. (2013). Experimental models of status epilepticus and neuronal injury for evaluation of therapeutic interventions. Int. J. Mol. Sci..

[3] Althaus A.L., Moore S.J., Zhang H., Du X., Murphy G.G., Parent J.M. (2019). Altered synaptic drive onto birthdated dentate granule cells in experimental temporal lobe epilepsy. J. Neurosci..

[4] Christiaen E., Goossens M.G., Raedt R., Descamps B., Larsen L.E., Craey E., Carrette E., Vonck K., Boon P., Vanhove C. (2019). Alterations in the functional brain network in a rat model of epileptogenesis: A longitudinal resting state fMRI study. Neuroimage.

[5] Kang T.C., Kim D.S., Kwak S.E., Kim J.E., Won M.H., Kim D.W., Choi S.Y., Kwon O.S. (2006). Epileptogenic roles of astroglial death and regeneration in the dentate gyrus of experimental temporal lobe epilepsy. Glia.

[6] Borges K., McDermott D., Irier H., Smith Y., Dingledine R. (2006). Degeneration and proliferation of astrocytes in the mouse dentate gyrus after pilocarpine-induced status epilepticus. Exp. Neurol..

[7] Kim J.E., Yeo S.I., Ryu H.J., Kim M.J., Kim D.S., Jo S.M., Kang T.C. (2010). Astroglial loss and edema formation in the rat piriform cortex and hippocampus following pilocarpine-induced status epilepticus. J. Comp. Neurol..

[8] Kim J.E., Ryu H.J., Kim M.J., Kim D.W., Kwon O.S., Choi S.Y., Kang T.C. (2010). Pyridoxal-5’-phosphate phosphatase/chronophin induces astroglial apoptosis via actin-depolymerizing factor/cofilin system in the rat brain following status epilepticus. Glia.

[9] Kim J.E., Kim Y.J., Kim J.Y., Kang T.C. (2014). PARP1 activation/expression modulates regional-specific neuronal and glial responses to seizure in a hemodynamic-independent manner. Cell Death Dis..

[10] Estrada F.S., Hernández V.S., López-Hernández E., Corona-Morales A.A., Solís H., Escobar A., Zhang L. (2012). Glial activation in a pilocarpine rat model for epileptogenesis: A morphometric and quantitative analysis. Neurosci. Lett..

[11] Bordey A., Sontheimer H. (1998). Properties of human glial cells associated with epileptic seizure foci. Epilepsy Res..

[12] Mathern G.W., Pretorius J.K., Kornblum H.I., Mendoza D., Lozada A., Leite J.P., Chimelli L., Born D.E., Fried I., Sakamoto A.C. (1998). Altered hippocampal kainate-receptor mRNA levels in temporal lobe epilepsy patients. Neurobiol. Dis..

[13] Represa A., Niquet J., Pollard H., Ben-Ari Y. (1995). Cell death, gliosis, and synaptic remodeling in the hippocampus of epileptic rats. J. Neurobiol..

[14] Kim D.S., Kim J.E., Kwak S.E., Choi K.C., Kim D.W., Kwon O.S., Choi S.Y., Kang T.C. (2008). Spatiotemporal characteristics of astroglial death in the rat hippocampo-entorhinal complex following pilocarpine-induced status epilepticus. J. Comp. Neurol..

[15] Kim J.E., Kwak S.E., Choi S.Y., Kang T.C. (2008). Region-specific alterations in astroglial TWIK-related acid-sensitive K+-1 channel immunoreactivity in the rat hippocampal complex following pilocarpine-induced status epilepticus. J. Comp. Neurol..

[16] Anderson C.M., Swanson R.A. (2000). Astrocyte glutamate transport: Review of properties, regulation, and physiological functions. Glia.

[17] Jäkel S., Dimou L. (2017). Glial cells and their function in the adult brain: A journey through the history of their ablation. Front. Cell. Neurosci..

[18] Lee S.H., Magge S., Spencer D.D., Sontheimer H., Cornell-Bell A.H. (1995). Human epileptic astrocytes exhibit increased gap junction coupling. Glia.

[19] Gabriel S., Kivi A., Kovacs R., Lehmann T.N., Lanksch W.R., Meencke H.J., Heinemann U. (1998). Effects of barium on stimulus-induced changes in [K^+^]o and field potentials in dentate gyrus and area CA1 of human epileptic hippocampus. Neurosci. Lett..

[20] Ricci G., Volpi L., Pasquali L., Petrozzi L., Siciliano G. (2009). Astrocyte-neuron interactions in neurological disorders. J. Biol. Phys..

[21] Takahashi D.K., Vargas J.R., Wilcox K.S. (2010). Increased coupling and altered glutamate transport currents in astrocytes following kainic-acid-induced status epilepticus. Neurobiol. Dis..

[22] Peixoto-Santos J.E., Velasco T.R., Galvis-Alonso O.Y., Araujo D., Kandratavicius L., Assirati J.A., Carlotti C.G., Scandiuzzi R.C., Santos A.C., Leite J.P. (2015). Temporal lobe epilepsy patients with severe hippocampal neuron loss but normal hippocampal volume: Extracellular matrix molecules are important for the maintenance of hippocampal volume. Epilepsia.

[23] Agarwal N.K., Mediratta P.K., Sharma K.K. (2011). Effect of lamotrigine, oxcarbazepine and topiramate on cognitive functions and oxidative stress in PTZ-kindled mice. Seizure.

[24] Tran T.A., McCoy M.K., Sporn M.B., Tansey M.G. (2008). The synthetic triterpenoid CDDO-methyl ester modulates microglial activities, inhibits TNF production, and provides dopaminergic neuroprotection. J. Neuroinflamm..

[25] Wang X.Y., Zhang X.H., Peng L., Liu Z., Yang Y.X., He Z.X., Dang H.W., Zhou S.F. (2017). Bardoxolone methyl (CDDO-Me or RTA402) induces cell cycle arrest, apoptosis and autophagy via PI3K/Akt/mTOR and p38 MAPK/Erk1/2 signaling pathways in K562 cells. Am. J. Transl. Res..

[26] Takagi T., Kitashoji A., Iwawaki T., Tsuruma K., Shimazawa M., Yoshimura S., Iwama T., Hara H. (2014). Temporal activation of Nrf2 in the penumbra and Nrf2 activator-mediated neuroprotection in ischemia-reperfusion injury. Free Radic. Biol. Med..

[27] Imai T., Takagi T., Kitashoji A., Yamauchi K., Shimazawa M., Hara H. (2016). Nrf2 activator ameliorates hemorrhagic transformation in focal cerebral ischemia under warfarin anticoagulation. Neurobiol. Dis..

[28] Kim J.E., Park H., Choi S.H., Kong M.J., Kang T.C. (2019). CDDO-Me selectively attenuates CA1 neuronal death induced by status epilepticus via facilitating mitochondrial fission independent of LONP1. Cells.

[29] Kim M.J., Park H., Choi S.H., Kong M.J., Kim J.E., Kang T.C. (2019). CDDO-Me attenuates vasogenic edema and astroglial death by regulating NF-κB p65 phosphorylations and Nrf2 expression following status epilepticus. Int. J. Mol. Sci..

[30] Park J.Y., Kang T.C. (2018). The differential roles of PEA15 phosphorylations in reactive astrogliosis and astroglial apoptosis following status epilepticus. Neurosci. Res..

[31] Kim J.E., Kang T.C. (2019). PKC, AKT and ERK1/2-mediated modulations of PARP1, NF-κB and PEA15 activities distinctly regulate regional specific astroglial responses following status epilepticus. Front. Mol. Neurosci..

[32] Kim J.E., Kang T.C. (2018). Nucleocytoplasmic p27(Kip1) export is required for ERK1/2-mediated reactive astroglial proliferation following status epilepticus. Front. Cell. Neurosci..

[33] Cragnolini A.B., Lampitella G., Virtuoso A., Viscovo I., Panetsos F., Papa M., Cirillo G. (2020). Regional brain susceptibility to neurodegeneration: What is the role of glial cells?. Neural Regen. Res..

[34] Culbreth M., Zhang Z., Aschner M. (2017). Methylmercury augments Nrf2 activity by downregulation of the Src family kinase Fyn. Neurotoxicology.

[35] Cuadrado A., Kügler S., Lastres-Becker I. (2018). Pharmacological targeting of GSK-3 and NRF2 provides neuroprotection in a preclinical model of tauopathy. Redox Biol..

[36] Park K.K., Liu K., Hu Y., Kanter J.L., He Z. (2010). PTEN/mTOR and axon regeneration. Exp. Neurol..

[37] Ross A.H., Gericke A. (2009). Phosphorylation keeps PTEN phosphatase closed for business. Proc. Natl. Acad. Sci. USA.

[38] Viatour P., Merville M.P., Bours V., Chariot A. (2005). Phosphorylation of NF-kappaB and IkappaB proteins: Implications in cancer and inflammation. Trends. Biochem. Sci..

[39] Duran A., Diaz-Meco M.T., Moscat J. (2003). Essential role of RelA Ser311 phosphorylation by zetaPKC in NF-kappaB transcriptional activation. EMBO J..

[40] Geng H., Wittwer T., Dittrich-Breiholz O., Kracht M., Schmitz M.L. (2009). Phosphorylation of NF-kappaB p65 at Ser468 controls its COMMD1-dependent ubiquitination and target gene-specific proteasomal elimination. EMBO Rep..

[41] Kim J.E., Kim D.S., Ryu H.J., Kim W.I., Kim M.J., Kim D.W., Choi S.Y., Kang T.C. (2013). The effect of P2X7 receptor activation on nuclear factor-κB phosphorylation induced by status epilepticus in the rat hippocampus. Hippocampus.

[42] Dinkova-Kostova A.T., Liby K.T., Stephenson K.K., Holtzclaw W.D., Gao X., Suh N., Williams C., Risingsong R., Honda T., Gribble G.W. (2005). Extremely potent triterpenoid inducers of the phase 2 response: Correlations of protection against oxidant and inflammatory stress. Proc. Natl. Acad. Sci. USA.

[43] Ahmad R., Raina D., Meyer C., Kharbanda S., Kufe D. (2006). Triterpenoid CDDO-Me blocks the NF-kappaB pathway by direct inhibition of IKKbeta on Cys-179. J. Biol. Chem..

[44] Yore M.M., Liby K.T., Honda T., Gribble G.W., Sporn M.B. (2006). The synthetic triterpenoid 1-[2-cyano-3,12-dioxooleana-1,9(11)-dien-28-oyl]imidazole blocks nuclear factor-kappaB activation through direct inhibition of IkappaB kinase beta. Mol. Cancer Ther..

[45] Konopleva M., Contractor R., Kurinna S.M., Chen W., Andreeff M., Ruvolo P.P. (2005). The novel triterpenoid CDDO-Me suppresses MAPK pathways and promotes p38 activation in acute myeloid leukemia cells. Leukemia.

[46] Correa F., Ljunggren E., Mallard C., Nilsson M., Weber S.G., Sandberg M. (2011). The Nrf2-inducible antioxidant defense in astrocytes can be both up- and down-regulated by activated microglia: Involvement of p38 MAPK. Glia.

[47] Ridet J.L., Malhotra S.K., Privat A., Gage F.H. (1997). Reactive astrocytes: Cellular and molecular cues to biological function. Trends. Neurosci..

[48] Horner P.J., Gage F.H. (2000). Regenerating the damaged central nervous system. Nature.

[49] Rossi D.J., Brady J.D., Morh C. (2007). Astrocyte metabolism and signaling during brain ischemia. Nat. Neurosci..

[50] Deeb D., Gao X., Dulchavsky S.A., Gautam S.C. (2007). CDDO-me induces apoptosis and inhibits Akt, mTOR and NF-kappaB signaling proteins in prostate cancer cells. Anticancer Res..

[51] Ling X., Konopleva M., Zeng Z., Ruvolo V., Stephens L.C., Schober W., McQueen T., Dietrich M., Madden T.L., Andreeff M. (2007). The novel triterpenoid C-28 methyl ester of 2-cyano-3, 12-dioxoolen-1, 9-dien-28-oic acid inhibits metastatic murine breast tumor growth through inactivation of STAT3 signaling. Cancer Res..

[52] Kulkarni A.A., Thatcher T.H., Olsen K.C., Maggirwar S.B., Phipps R.P., Sime P.J. (2011). PPAR-γ ligands repress TGFβ-induced myofibroblast differentiation by targeting the PI3K/Akt pathway: Implications for therapy of fibrosis. PLoS ONE.

[53] Liu Y., Gao X., Deeb D., Gautam S.C. (2012). Oleanane triterpenoid CDDO-Me inhibits Akt activity without affecting PDK1 kinase or PP2A phosphatase activity in cancer cells. Biochem. Biophys. Res. Commun..

[54] Hollander M.C., Blumenthal G.M., Dennis P.A. (2011). PTEN loss in the continuum of common cancers, rare syndromes and mouse models. Nat. Rev. Cancer.

[55] Bunney T.D., Katan M. (2010). Phosphoinositide signalling in cancer: Beyond PI3K and PTEN. Nat. Rev. Cancer.

[56] Vanhaesebroeck B., Guillermet-Guibert J., Graupera M., Bilanges B. (2010). The emerging mechanisms of isoform-specific PI3K signalling. Nat. Rev. Mol. Cell Biol..

[57] Mello L.E., Cavalheiro E.A., Tan A.M., Kupfer W.R., Pretorius J.K., Babb T.L., Finch D.M. (1993). Circuit mechanisms of seizures in the pilocarpine model of chronic epilepsy: Cell loss and mossy fiber sprouting. Epilepsia.

[58] Holmes G.L., Sarkisian M., Ben-Ari Y., Chevassus-Au-Louis N. (1999). Mossy fiber sprouting after recurrent seizures during early development in rats. J. Comp. Neurol..

[59] Revuelta M., Castano A., Machado A., Cano J., Venero J.L. (2005). Kainate-induced zinc translocation from presynaptic terminals causes neuronal and astroglial cell death and mRNA loss of BDNF receptors in the hippocampal formation and amygdale. J. Neurosci. Res..

[60] Toscano E.C.B., Vieira É.L.M., Portela A.C.D.C., Reis J.L.J., Caliari M.V., Giannetti A.V., Gonçalves A.P., Siqueira J.M., Suemoto C.K., Leite R.E.P. (2019). Bcl-2/Bax ratio increase does not prevent apoptosis of glia and granular neurons in patients with temporal lobe epilepsy. Neuropathology.

[61] Chiarugi A. (2002). Characterization of the molecular events following impairment of NF-kappaB-driven transcription in neurons. Brain Res. Mol. Brain Res..

[62] Kim J.E., Ryu H.J., Yeo S.I., Kang T.C. (2011). P2X7 receptor differentially modulates astroglial apoptosis and clasmatodendrosis in the rat brain following status epilepticus. Hippocampus.

[63] Wang C.C., Fang K.M., Yang C.S., Tzeng S.F. (2009). Reactive oxygen species-induced cell death of rat primary astrocytes through mitochondria-mediated mechanism. J. Cell. Biochem..

[64] Dinkova-Kostova A.T., Holtzclaw W.D., Cole R.N., Itoh K., Wakabayashi N., Katoh Y., Yamamoto M., Talalay P. (2002). Direct evidence that sulfhydryl groups of KEAP1 are the sensors regulating induction of phase 2 enzymes that protect against carcinogens and oxidants. Proc. Natl. Acad. Sci. USA.

[65] Chen X.L., Kunsch C. (2004). Induction of cytoprotective genes through Nrf2/antioxidant response element pathway: A new therapeutic approach for the treatment of inflammatory diseases. Curr. Pharm. Des..

[66] Kobayashi A., Kang M.I., Watai Y., Tong K.I., Shibata T., Uchida K., Yamamoto M. (2006). Oxidative and electrophilic stresses activate Nrf2 through inhibition of ubiquitination activity of Keap1. Mol. Cell. Biol..

[67] Liby K., Royce D.B., Williams C.R., Risingsong R., Yore M.M., Honda T., Gribble G.W., Dmitrovsky E., Sporn T.A., Sporn M.B. (2007). The synthetic triterpenoids CDDO-methyl ester and CDDO-ethyl amide prevent lung cancer induced by vinyl carbamate in A/J mice. Cancer Res..

[68] Murphy T.H., Yu J., Ng R., Johnson D.A., Shen H., Honey C.R., Johnson J.A. (2001). Preferential expression of antioxidant response element mediated gene expression in astrocytes. J. Neurochem..

[69] Johnson D.A., Andrews G.K., Xu W., Johnson J.A. (2002). Activation of the antioxidant response element in primary cortical neuronal cultures derived from transgenic reporter mice. J. Neurochem..

[70] Shih A.Y., Johnson D.A., Wong G., Kraft A.D., Jiang L., Erb H., Johnson J.A., Murphy T.H. (2003). Coordinate regulation of glutathione biosynthesis and release by Nrf2-expressing glia potently protects neurons from oxidative stress. J. Neurosci..

[71] Guizzetti M., Bordi F., Dieguez-Acuña F.J., Vitalone A., Madia F., Woods J.S., Costa L.G. (2003). Nuclear factor kappaB activation by muscarinic receptors in astroglial cells: Effect of ethanol. Neuroscience.

[72] Yang C.C., Lin C.C., Jou M.J., Hsiao L.D., Yang C.M. (2019). RTA 408 inhibits interleukin-1β-induced MMP-9 expression via suppressing protein kinase-dependent NF-κB and AP-1 activation in rat brain astrocytes. Int. J. Mol. Sci..

[73] Buss H., Dörrie A., Schmitz M.L., Frank R., Livingstone M., Resch K., Kracht M. (2004). Phosphorylation of serine 468 by GSK-3beta negatively regulates basal p65 NF-kappaB activity. J. Biol. Chem..

[74] Meng X., Waddington J.C., Tailor A., Lister A., Hamlett J., Berry N., Park B.K., Sporn M.B. (2020). CDDO-imidazolide targets multiple amino acid residues on the Nrf2 adaptor, Keap1. J. Med. Chem..

