# Supplementary material for: CDDO-Me Distinctly Regulates Regional Specific Astroglial Responses to Status Epilepticus via ERK1/2-Nrf2, PTEN-PI3K-AKT and NFκB Signaling Pathways
Source: Antioxidants (Basel). 2020 Oct 21;9(10):1026. doi: 10.3390/antiox9101026 (PMC7589507; doi:10.3390/antiox9101026)
Supplement: Supplementary file 1 [file antioxidants-09-01026-s001.pdf]

SUPPORTING INFORMATIONS

CDDO-Me distinctly regulates regional specific astroglial responses to status epilepticus via ERK1/2-Nrf2, PTEN-PI3K-AKT and NFκB signaling pathways

Ji-Eun Kim[1,2], Hana Park[1,2], Tae-Cheon Kang[1,2*]

[1]Department of Anatomy and Neurobiology, College of Medicine, Hallym University, Chuncheon 24252, South Korea

[2]Institute of Epilepsy Research, College of Medicine, Hallym University, Chuncheon 24252, South Korea

* Correspondence to: T. -C. Kang, Department of Anatomy and Neurobiology, College of Medicine, Hallym University, Chuncheon, Kangwon-Do 24252, South Korea; Tel: +82-33-248-2524; Fax: +82-33-248-2525; E-mail: tckang@hallym.ac.kr

Fig. 1A

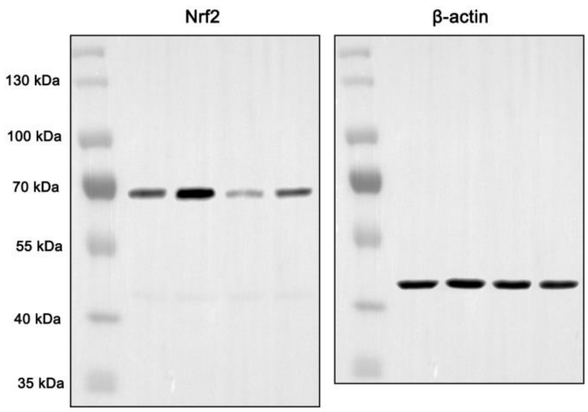

Fig. 3A

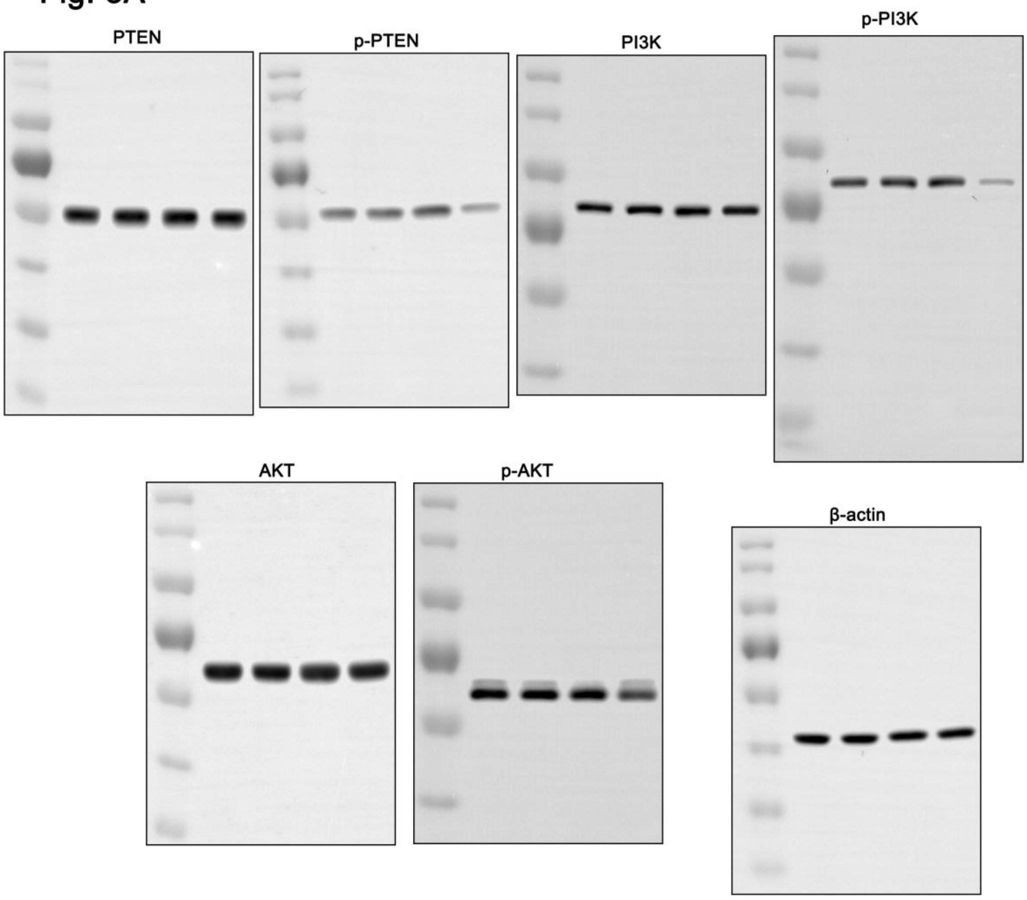

Supplementary Figure 1. Full-gel images of Western blots in Fig. 1A and 3A.

Fig. 6A

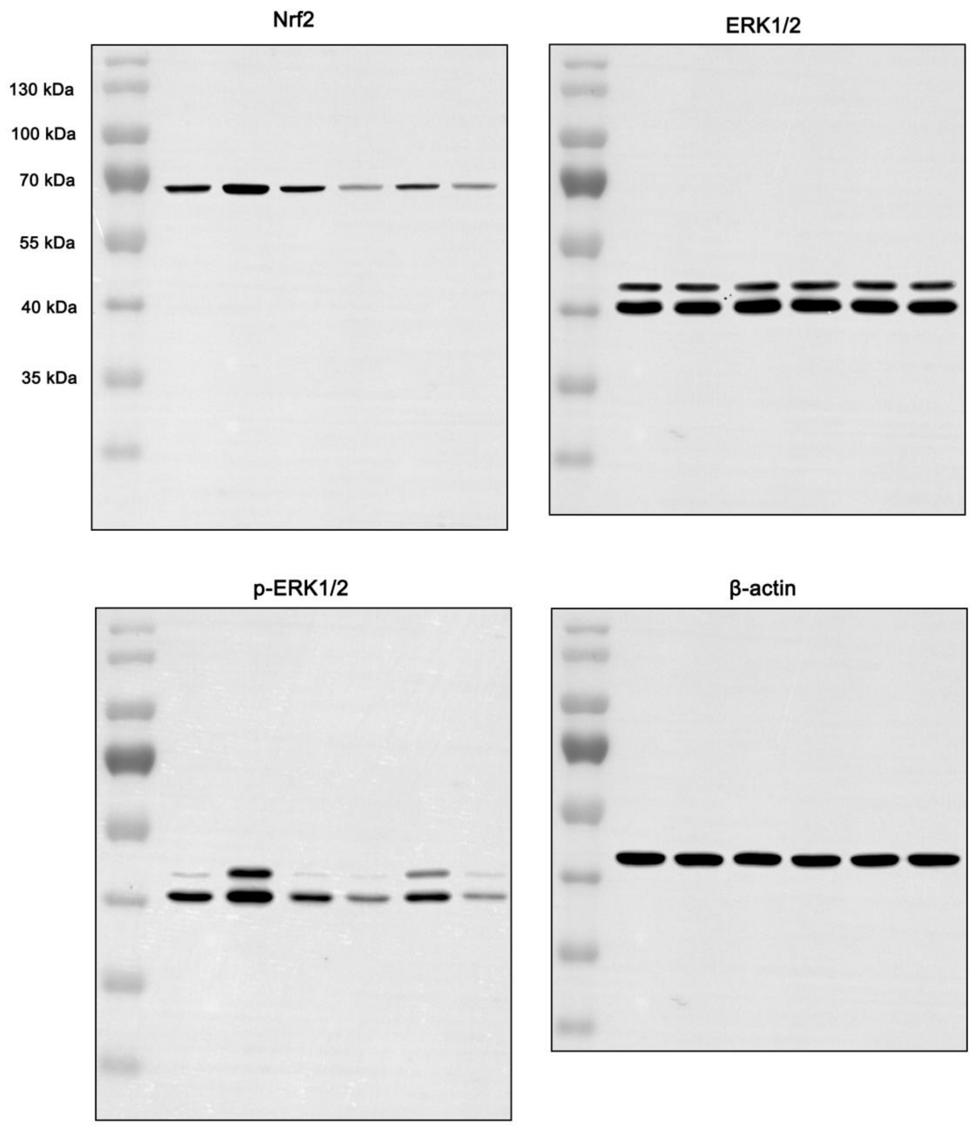

Supplementary Figure 2. Full-gel images of Western blots in Fig. 6A.
